# Biological Control Options for the Golden Twin-Spot Moth, *Chrysodeixis chalcites* (Esper) (Lepidoptera: Noctuidae) in Banana Crops of the Canary Islands

**DOI:** 10.3390/insects13060516

**Published:** 2022-05-31

**Authors:** Modesto del Pino, Tomás Cabello, Estrella Hernández-Suárez

**Affiliations:** 1 Laboratory of Agricultural Entomology, Andalusian Institute for Research and Training in Agriculture, Fishery, Food and Ecological Production (IFAPA), Málaga Centre, Cortijo de la Cruz s/n, Churriana, 29140 Malaga, Spain; modesto.pino@juntadeandalucia.es; 2 Center for Agribusiness Biotechnology Research, University of Almeria (UAL), 04120 Almería, Spain; tcabello@ual.es; 3 Department of Plant Protection, Canary Institute of Agricultural Research (ICIA), 38200 La Laguna, Spain

**Keywords:** tomato looper, *Trichogramma achaeae*, parasitoid, survey, parasitism, biological control, integrated pest management

## Abstract

**Simple Summary:**

The golden twin-spot moth, *Chrysodeixis chalcites*, is a pest whose larvae cause serious skin injuries to banana fruits in the Canary Islands, reducing their commercial value. The use of Integrated Pest Management (IPM) strategies (cultural, biological, and chemical control) is recommended for the effective and sustainable management of this pest. The identification of its parasitoids and the quantification of their effects on pest populations are essential for the development of biological controls. In this study, we conducted an extensive survey to identify the most important parasitoid species of *Ch. chalcites* and evaluated the efficacy of the egg parasitoid *Trichogramma achaeae* as a biological control agent in banana plantations in the Canary Islands. Our findings indicate that populations of native parasitoids can exert a certain degree of natural control over *Ch. chalcites*. However, their naturally occurring populations are insufficient to minimize the damage caused by this pest. Thus, the development of IPM programs based on the use of selective insecticides, the conservation of natural enemies and inundative releases of mass-reared wasps is also necessary. The parasitoid *T. achaeae* has been identified as a promising biological control agent of *Ch. chalcites* in greenhouse banana crops, but it is necessary to carry out further studies to establish the most appropriate release strategies.

**Abstract:**

*Chrysodeixis chalcites* (Esper) (Lepidoptera: Noctuidae) is a significant pest in banana plantations in the Canary Islands. Field surveys were carried out to identify its naturally occurring parasitoids and estimate their parasitism rates between September 2007 and October 2010. *Ch. chalcites* was parasitized by six different larval/pupal parasitoid species: *Cotesia* sp., *C. glomerata* L. (Hym.: Braconidae), *Aplomyia confinis* Fallén (Dip.: Tachinidae), *Hyposoter rufiventris* Perez, *Ctenochares bicolorus* L. (Hym.: Ichneumonidae) and *Aleiodes* sp. (Hym.: Braconidae). Among them, *Cotesia* sp. was the most frequent species, accounting for 8.18% of parasitized larvae. High levels of egg parasitism were detected, with *Trichogramma achaeae* Nagaraja and Nagarkatti (Hym.: Trichogrammatidae) being the most widely distributed egg parasitoid. A greenhouse assay was also carried out on a commercial banana crop with the aim of evaluating the potential of *T. achaeae* as a biological control agent and compared with a chemical control. Five periodic inundative releases of 35 adults/m^2^ every 21 days were necessary to achieve an adequate parasitism level (56.25 ± 1.61%). Moreover, there was 15.75% less foliar damage in the biological control plot compared to the chemical control plot. These results indicate that *T. achaeae* could be a promising biocontrol agent of *Ch. chalcites* in greenhouse banana crops.

## 1. Introduction

Banana (*Musa acuminata* Colla) is a tropical plant species native to Southern Asia belonging to the Musaceae family, which is grown commercially in tropical and subtropical regions of the world [1]. Currently, banana is among the most produced, traded and consumed fresh fruits worldwide with a global production that reached more than 119 million tons in 2020 [2]. The Canary Islands (Spain) are the main producer of the European Union with a stable production area of around 9000 ha and total banana production of about 0.43 million tons in 2020 [3]. Most of the banana production is for the markets of mainland Spain with the rest sold locally [1].

Banana crops present numerous phytosanitary problems caused by the special climatic conditions in the Canary Island archipelago [4]. Among them, the golden twin spot moth or tomato looper, *Chrysodeixis chalcites* (Esper) (Lepidoptera: Noctuidae), stand out due to the serious skin injuries that its larvae cause when they feed on developing fruits, which subsequently reduces these fruits’ commercial value [5]. Crop losses can affect up to 5.7% of the banana production grown under mesh-greenhouse conditions if the control measures are not effective [6]. This subtropical and polyphagous species occurs in many regions of Europe, the Mediterranean, the Middle East, and Africa, where its larvae feed on many fruits, as well as horticultural and ornamental cultivated plants [7]. In the Canary Islands, *Ch. chalcites* is a polyvoltine species that occurs throughout the banana production cycle, with up to seven or eight overlapping generations per year, with the two predominant flights in spring (May–June) and autumn (September–October) [5,8]. The larvae of *Ch. chalcites* feed preferentially on young plants; however, the most sensitive growing periods are from transplanting to the first foliar stages and flowering and fruiting [6], which occur during the main peak flight in the spring [1,8].

The control of *Ch. chalcites* is mainly based on multiple applications of a low number of active substances authorized by the European Union throughout the crop cycle [9]. However, these may increase the risks associated with the development of pesticide resistance [10] and have negative impacts on beneficial insects and the environment [11], as well as generating pesticide residues that hinder the commercialization of the fruit [12]. Therefore, it is necessary to develop Integrated Pest Management (IPM) strategies in accordance with current European Union regulations for the sustainable use of pesticides (European Directive, 2009/128/EEC), based on the conservation of natural enemies and application of low-risk insecticides, which would help minimize economic losses caused by *Ch. chalcites* in banana plantations [13,14,15]. 

Knowledge of the role of natural enemies is a key factor in the development of IPM programs [16]. Their identification and the quantification of their effects on pest populations are essential for an effective biological control of pests [17], especially when the introduction of exotic natural enemies is complicated by biological, ecological, or economic factors [18,19]. Studies on biological control of *Ch. chalcites* are limited, perhaps due to the lower economic interest of this pest compared to other Lepidoptera species [20]. However, a variety of parasitoids and predators have been reported [21,22,23,24,25] and evaluated as biological control agents for augmentative control of *Ch. chalcites* [26,27,28], although most of them are not yet commercially available [20]. 

In the Macaronesian region, some native natural enemies have been found to be associated with *Ch. chalcites* populations. Generalist predators such as *Chrysoperla carnea* Stephens (Neuroptera: Chrysopidae) and different species of spiders are usually present in banana crops [4]. As for parasitoids, several species belonging to the genus *Trichogramma* (Hymenoptera: Trichogrammatidae) have been recorded parasitizing *Ch. chalcites* eggs in Madeira, Azores, and Canary Islands [29,30,31,32]. Among them, *T. achaeae* Nagaraja and Nagarkatti has been reported as the most important species in the main banana-producing islands of the Canary archipelago [33], showing high potential as a biological control agent [34]. However, no studies have been published yet on the potential *T. achaeae* releases to suppress *Ch. chalcites* populations in banana plantations. Furthermore, some species of larval/pupal parasitoids belonging to Braconidae, Eulophidae, Ichneumonidae and Tachinidae families have also been reported [21,35,36,37]. Regarding entomopathogens, an alphabaculovirus of *Ch. chalcites* (ChchNPV-TF1) has been isolated from banana plantations in southern Tenerife [38] and evaluated as an effective bioinsecticide in laboratory and field conditions [39,40].

The present study is aimed at conducting an extensive survey in the main banana production areas of the Canary Islands and for a broad period of time, to (1) identify the most important parasitoid species of *Ch. chalcites* and other noctuid pests attacking banana crops; (2) determine the rate of parasitism for each parasitoid species; (3) identify those that have the greatest effect on pest populations; and (4) evaluate the efficacy of *T. achaeae* as biological control agent of *Ch. chalcites* in banana plantations. The overarching aim is the development of IPM strategies for *Ch. chalcites* in the banana plantations of the Canary Islands.

## 2. Materials and Methods

### 2.1. Natural Parasitism

A total of 177 field surveys were carried out to determine the occurrence of parasitoids in 59 commercial banana plantations grown in open-field and mesh-built greenhouses between September 2007 and October 2010, in the four major banana-producing islands (Tenerife, La Palma, Gran Canaria and El Hierro) of the archipelago (Table S1). Parasitoids were obtained from eggs and larvae of *Ch. chalcites* and other noctuid species randomly collected in banana and non-crop plants: tomato (*Solanum lycopersicum* L.), squash (*Cucurbita maxima* Duch. ex Lam.), cabbage (*Brassica oleracea* L. var. viridis), sweet pepper (*Capsicum annuum* L.), green beans (*Phaseolus vulgaris* L.), geranium (*Pelargonium* spp. L’Hér.), *Nicotiana glauca* Graham and *Solanum nigrum* L.

In each plantation, banana plants and non-crop plants were randomly surveyed for 1 h, collecting larvae and leaves with *Ch. chalcites* eggs into paper bags. Collected eggs and larvae were transported to the laboratory and individually stored in glass tubes (5 × 1 cm) or plastic vials (25 mL), respectively, until the emergence of adult wasps. Collected larvae were provided with a semi-synthetic diet commonly used to rear *Ch. chalcites* and other polyphagous lepidopteran species [5]. Diet was replaced every two days. In both cases, eggs and larvae were examined daily and reared in a climatic chamber (25 ± 1 °C, 65 ± 10 R.H. and 16:8 h of L:D) until moth adult or parasitoid emergence. Adult parasitoids were preserved in 70% ethanol in 1.5 mL vials and labelled for later identification at the species level, where possible, with stereoscope microscopy. Egg parasitoids were morphologically and molecularly identified according to the methodology described in Polaszek et al. [31] and del Pino et al. [32,33]. Larval parasitoid species were morphologically identified by Andrew Polaszek (Natural History Museum, London, UK) and Gloria Ortega (Natural Sciences Museum, Tenerife, Spain).

### 2.2. Efficacy of T. achaeae as Biological Control Agent

#### 2.2.1. Experimental Design

To evaluate the efficacy of *T. achaeae* as a biological control agent of *Ch. chalcites*, a trial was carried out in a mesh-built greenhouse (1 ha) with a young commercial banana crop cultivar Gran Enana located in Fañabé (Adeje, Tenerife) (28°6′26.54″ N; 16°3′13.02″ W; altitude 18 m) from August to December 2010. Banana seedling plants were transplanted at the end of July using a plantation framework of 1.8 m between holes and 2.0 m between rows. Two plants were planted per hole. Plants were drip-irrigated and maintained following cultural practices routinely used in this banana cultivation area. The greenhouse was divided into two modules (3500 m^2^) without physical separation. One of the modules was used as a control plot, receiving only chemical treatments, following the common practice used by the growers. In the second module, *T. achaeae* was released at a dosage of 35 adults/m^2^ using dispensing cards (TrichoControl©) supplied by AgroBio S.L. (Almeria, Spain) and homogeneously placed throughout the plot by inserting them on the last leaf of the stem of the plants. The release dose was established from those previously used in the biological control programs of *T. absoluta* in tomato crops in greenhouses [41]. There was a total of five releases of *T. achaeae* every 21 or 28 days on the following days: 16 August; 6 and 27 September; 25 October and 16 November 2010. Each release contained a single cohort of adults, which emerged at 12–24 h after field release. The following insecticide treatments were applied to the chemical plot during the study period: Chlorpyrifos (Dursban 48% EC, Dow Agrosciences Iberica, Madrid, Spain; 2 mL/liter) on 10 September, and *B. thuringiensis* subsp. *kurstaki* (Sequra 32% WG, Kenogard, Barcelona, Spain; 1.0 g/liter) on 24 September. Each main plot was divided into four blocks or elementary plots (subplot), consisting of 6 paired crop lines with 8 plants each. The plants were naturally infested with *Ch. chalcites*.

#### 2.2.2. Sampling

Every week, ten randomly selected banana plants were sampled per block, always from the central rows, with the rest acting as borders. In each plant, the first five young leaves recently expanded in the upper part of the plant were sampled, because it is the favorite plant section for oviposition of *Ch. chalcites* females [42]. On each leaf, the number of eggs (total and parasitized), larvae, and pupae of *Ch. chalcites*, as well as the level of damage caused by the larvae, were directly quantified. Foliar damage was evaluated in banana plants using a visual scale with five categories [6]: Category 0: no damage, Category 1: 5–20% leaf damage (percentage of leaf area showing damage), Category 2: 21–40% leaf damage, Category 3: 41–60% leaf damage and Category 4: >60% leaf damage. Thereafter, the average percentage severity of foliar damage was calculated using the formula described by Townsend and Heuberger [43]:

(1) $$\sum \left(\frac{n\times v}{V\times N}\right)\times 100$$

where *n* is the number of sample units in each category, *v* the value of each category (0, 1, 2, 3 or 4), *V* the value of the highest category and *N* the total number of sample units. Temperature and relative humidity values were monitored by means of a Data Logger (HOBO^®^ Pro v2 Data Logger, Onset Computer Corporation, Bourne, MA, USA) placed inside the greenhouse. During the trial, the average, maximum, and minimum temperature values were 22.8 °C, 38.5 °C, and 12.3 °C, respectively.

### 2.3. Statistics

Data are expressed as mean values and standard errors (±SE). In the natural parasitism surveys, data obtained for the number of *Ch. chalcites* eggs (sampled and parasitized) and the percentage of parasitism per field were analyzed using a one-way ANOVA test and the GLM procedure. Average values were compared with Tukey’s test (*p* = 0.05) by means of the R-based program Jamovi v.2.2. [44]. In the greenhouse assay, pest density data (eggs and larvae) were normalized by Ln(x + 1) transformation, while parasitism and damaged area rate data were transformed to arcsin √(x/100). All transformed data were subjected to *t*-tests, using the same program.

## 3. Results

### 3.1. Natural Parasitism

From 2007 to 2010, a total of 2776 eggs of *Ch. chalcites* were collected from naturally infested plants, of which 64.30% (*n* = 1785) were parasitized (Table 1). Egg parasitism varied from one island to another. The highest natural parasitism was registered on La Palma, with 90.06% (*n* = 988), followed by El Hierro, Gran Canaria and Tenerife, with 65.57% (*n* = 40), 54.84% (*n* = 136) and 48.76% (*n* = 668), respectively. Furthermore, in the surveys carried out, numerous hatched parasitized eggs were found on banana and non-crop vegetation leaves. 

Average egg parasitism per field varied significantly between islands (*F* = 8.647; *df* = 3; *p* < 0.001), with the highest being in La Palma (81.01 ± 6.36%), followed by Gran Canaria (70.82 ± 21.08%), El Hierro (48.57 ± 11.27%) and Tenerife (38.58 ± 5.63), reaching maximum peaks of between 71.64 and 100.00% (Table 2). 

According to morphological and molecular identification, all individuals that emerged from the eggs of *Ch. chalcites* belonged to five species of the genus *Trichogramma* (Hymenoptera: Trichogrammatidae): *T. achaeae* Nagaraja and Nagarkatti, *T. bourarachae* Pintureau and Babault, *T. canariensis* del Pino and Polaszek, *T. euproctidis* Guirault and *T. evanescens* Westwood. *T. achaeae* has been the most widely distributed species and has been found throughout all islands.

The total number of noctuid species, mortality percentages (mostly due to ChchNPV-TF1) and larval/pupal parasitism on the four sampled islands are shown in Table 3. A total of 5922 noctuid larvae were collected during the survey from 2007 to 2010, belonging to five species: *Ch. chalcites*, *Spodoptera littoralis* (Boisdusal, 1833), *S. exigua* (Hübner, 1808), *Cornutiplusia circunflexa* (Linnaeus, 1767) and *Trichoplusia orichalcea* (Fabricius, 1775). Most of the collected larvae came from banana plants, where higher infestations were found. *Ch. chalcites* was the most abundant species representing 92.52% (*n* = 5479) of the total collected larvae, followed by *S. littoralis* with 4.86% (*n* = 288) and *C. circunflexa* with 1.60% (*n* = 95). A total of 285 parasitoids emerged from the collected larvae, which represented 6.98% of the total number of samples. The number of parasitized larvae maintained a close correlation with the relative abundances found in natural populations of each species; thus, of the 285 parasitized larvae, 94.04% (*n* = 268) were *Ch. chalcites*, 3.51% (*n* = 10) of *C. circunflexa* and 2.46% (*n* = 7) of *S. littoralis*. There was 36.96% (*n* = 2025) of *Ch. chalcites* larvae that died by parasitism (*n* = 268) or for other reasons (*n* = 1757). Mortality by infectious diseases or undetermined causes was 32.07% of the total collected larvae.

The levels of parasitism per field for each larval/pupal parasitoid species, host species and plant species are shown in Table 4. *Ch. chalcites* was parasitized by six different larval/pupal parasitoid species: *Cotesia* sp., *C. glomerata* L. (Hym.: Braconidae), *Aplomyia confinis* Fallén (Dip.: Tachinidae), *Hyposoter rufiventris* Perez, *Ctenochares bicolorus* L. (Hym.: Ichneumonidae) and *Aleiodes* sp. (Hym.: Braconidae). Among them, *Cotesia* sp. was the most frequent species, occurring in 8.18% of the cases, followed by *C. bicolorus* (4.60%), *A. confinis* (4.22%), *C. glomerata* (3.01%) and *H. rufiventris* (2.18%). Some parasitoids showed a degree of host specificity, for example, *A. confinis* was only found parasitizing *Ch. chalcites* larvae in geranium and banana plants, although the average level of parasitism was higher in geranium (5.26%) than in banana (4.22%). On the other hand, *Cotesia* sp. and *H. rufiventris* were found attacking different noctuid hosts. 

Table 5 summarizes *Ch. chalcites* parasitoid species found on the four surveyed islands from 2007 to 2010. Adult parasitoids were obtained from banana, tomato, *N. glauca* and *S. nigrum* plants in all surveyed areas. These records suggest a higher diversity of parasitoid species in locations characterized by the presence of abundant non-crop vegetation; 10 species have been found in Tenerife, 6 in La Palma, 4 in El Hierro and 2 in Gran Canaria islands. In total, eleven different species were reared from eggs and larvae belonging to four different families: Trichogrammatidae, Braconidae, Ichneumonidae and Tachinidae, but two of these have only been identified to genus level. 

### 3.2. Efficacy of T. achaeae as Biological Control Agent

**Figure 1:**
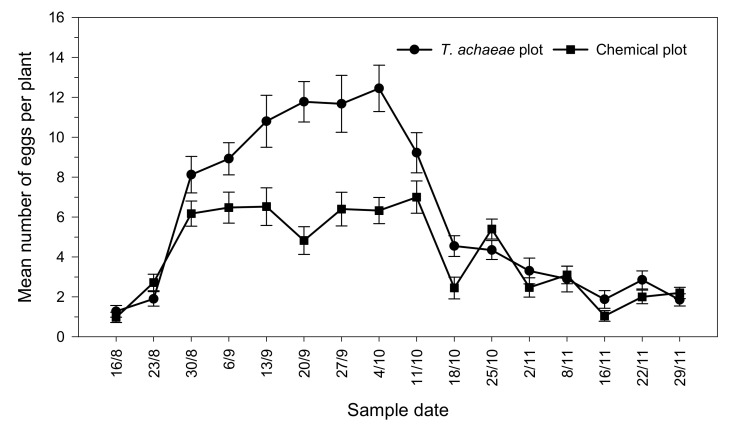
Progression of the mean number (±SE) of *Ch. chalcites* eggs per plant in greenhouse banana crop according to treatment (*Trichogramma achaeae* releases compared with chemical control).

Figure 1 shows the evolution of the mean number of *Ch. chalcites* eggs found per plant, according to treatment, on the different sampling dates. At the beginning of the trial, there were no significant differences between the chemical and biological control plots (*t* = 0.919; *df* = 78; *p* = 0.740). However, the egg density subsequently increased to a maximum of 12.45 ± 1.16 eggs per plant (October 4) in the plot with *T. achaeae* releases and 7.00 ± 0.81 eggs per plant (October 11) in the chemical control plot. 

**Figure 2:**
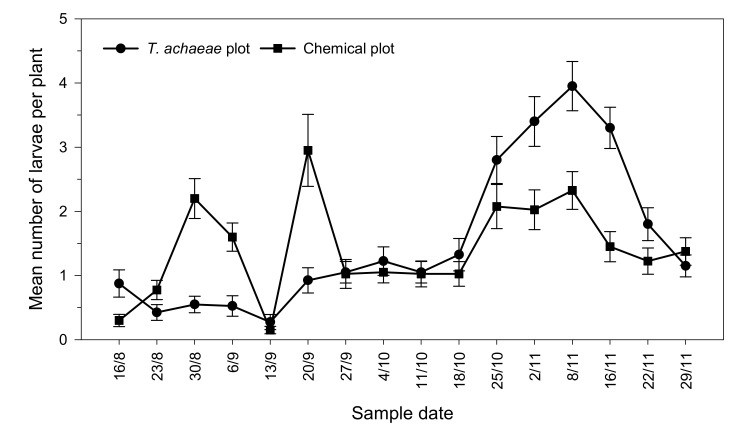
Progression of the mean number (±SE) of *Ch. chalcites* larvae per plant in greenhouse banana crop according to treatment (*Trichogramma achaeae* releases compared with chemical control).

The evolution of the population level of *Ch. chalcites* larvae is shown in Figure 2. At the beginning of the trial, significant differences were found between both plots (*t* = 2.345; *df* = 78; *p* < 0.001), with larvae population being higher in the biological control plot (0.88 ± 0.21 larvae/plant) than in the chemical control plot (0.30 ± 0.10 larvae/plant). However, the number of larvae per plant increased to 2.20 ± 0.31 (August 30) and 2.95 ± 0.56 (September 20) in the chemical control plot, which forced the application of Chlorpyrifos and *B. thuringiensis* subsp. *kurstaki* at commercial doses on September 10 and 24, respectively. In the case of the *T. achaeae* release plot, the number of larvae per plant remained stable, but at the end of the trial increased to average values of 3.95 ± 0.38 larvae per leaf (8 November).

**Figure 3:**
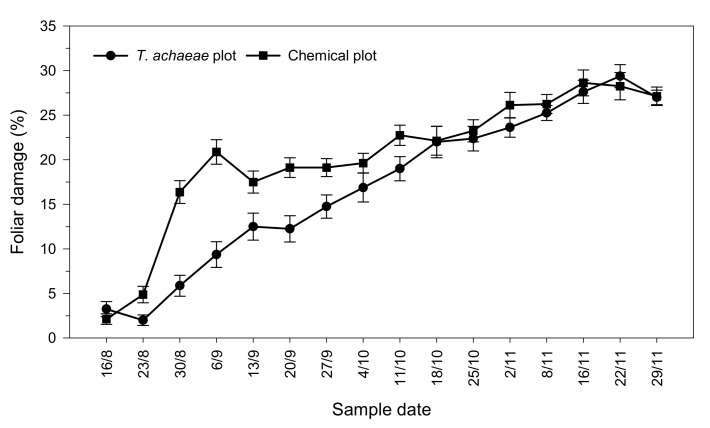
Progression of foliar damage (± SE) by *Ch. chalcites* in greenhouse banana crop according to treatment (*Trichogramma achaeae* releases compared with chemical control).

Concerning foliar damage, no significant differences were found (*t* = 1.033; *df* = 78; *P* = 0.101) between plots at the beginning of the trial. However, damage increased significantly from the second release (*t* = -2.610; *df* = 78; *p* < 0.001), reaching the highest foliar damage at the end of the trial with mean values of 27.13 ± 1.03% for the chemical control plot and 27.00 ± 0.82% for the biological control plot (Figure 3).

**Figure 4:**
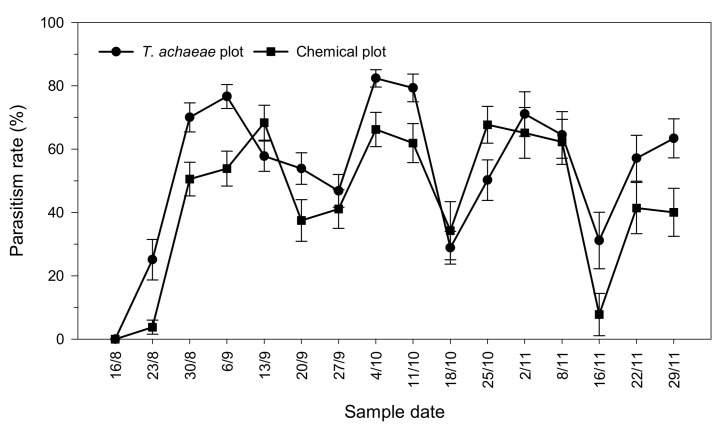
Progression of parasitism rate (± SE) in greenhouse banana crop according to treatment (*Trichogramma achaeae* releases compared with chemical control).

Mean parasitism rate showed no significant differences between plots at the beginning of the trial; in fact, no natural parasitism was detected in the crop (Figure 4). However, after the first release of *T. achaeae*, significant differences were found in the second week (*t* = 3.582; *df* = 56; *p* < 0.001). The maximum percentage of parasitized *Ch. chalcites* eggs was 82.36 ± 2.73% (4 October) in the plot with *T. achaeae* releases and 68.35 ± 5.52% (13 September) in the chemical control plot. From 13 to 27 September, there was a marked reduction in the parasitism rate, mainly due to the low emergence of *T. achaeae* adults from dispensing cards in the second release (9 September). However, parasitism recovered satisfactorily after the third release (27 September).

**Figure 5:**
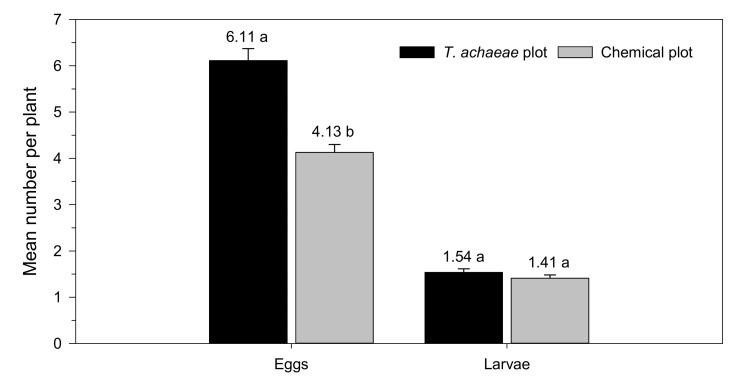
Mean number (± SE) of eggs and larvae of *Ch. chalcites* per plant according to treatment (*Trichogramma achaeae* releases compared with chemical control). Values followed by different letters denote significant differences among treatments (*t*-tests, *p* ≤ 0.05).

Figure 5 shows the effect of *T. achaeae* releases on the population size of *Ch. chalcites*, measured by the mean number of eggs and larvae per plant for the period of the study. The mean number of eggs per plant was significantly higher in the plot with *T. achaeae* releases than in the chemical control plot (*t* = 4.964; *df* = 1278; *p* < 0.001), with 6.11 ± 0.27 and 4.13 ± 0.17 eggs per plant, respectively. However, the mean number of larvae per plant was similar in both plots (*t* = 0.970; *df* = 1278; *p* = 0.332), with 1.54 ± 0.08 and 1.41 ± 0.07 larvae per plant in *T. achaeae* releases and chemical control plots, respectively. 

**Figure 6:**
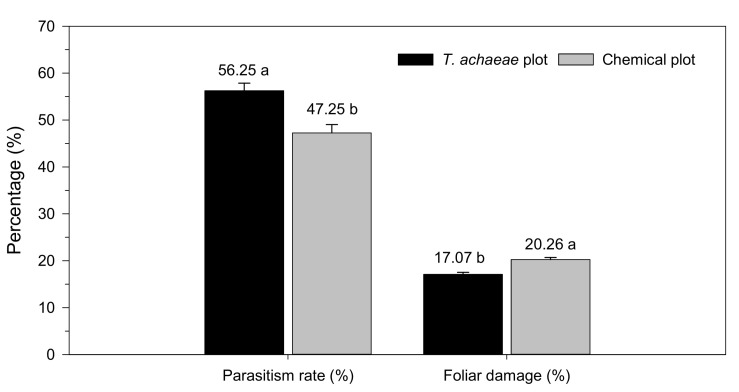
Mean parasitism rate (± SE) and mean foliar damage (± SE) according to treatment (*Trichogramma achaeae* releases compared with chemical control). Values followed by different letters denote significant differences among treatments (*t*-tests, *p* ≤ 0.05).

Furthermore, there were differences between treatments in the percentage of foliar damage (*t* = -5.546; *df* = 1278; *p* < 0.001), with higher damage in the chemical control plot (20.26 ± 0.42%) than in the biological control plot (17.07 ± 0.47%) (Figure 6). These results indicate that foliar damage in the biological control plot was 1.19 times less (15.75%) than in the control plot. Finally, the parasitism rate was significantly higher (*t* = 3.874; *df* = 1020; *p* < 0.001) in the biological control plot, with an average value of 56.25 ± 1.61% with respect to that recorded in the chemical plot, which was 47.25 ± 1.81%.

## 4. Discussion

The results from the survey of naturally occurring parasitoids of *Ch. chalcites*, carried out in a variety of growing habitats (open field and mesh-built greenhouse), have provided novel and relevant information contributing to knowledge of this key pest in banana plantations on the Canary Islands. Eleven parasitoid species were recorded attacking *Ch. chalcites*, of which eight were found over a period longer than a year, suggesting a repeated use of this noctuid pest as host (Table 5). They belonged to the following families: Trichogrammatidae, Braconidae, Ichneumonidae and Tachinidae.

Several studies have been carried out in the past on the natural enemies of *Ch. chalcites* from different regions. For example, a total of 14 parasitoid species have been found in banana crops of Guinea and Ivory Coast [21]. Lower parasitoid richness has been detected in outdoor crops [22,45] and greenhouse crops [46] on mainland Spain. Overall, almost 30 different taxa of parasitoids have been detected exploiting *Ch. chalcites* as a host in the Macaronesia and Mediterranean regions [7]. These parasitoids comprise eight Ichneumonidae, eight Trichogrammatidae, five Braconidae, three Encyrtidae, two Eulophidae, two Scelionidae, one Chalcididae and one Tachinidae. 

All egg parasitoids found in the present study belonged to the *Trichogramma* genus, with the highest natural parasitism rates per field on the islands of La Palma (81.01%) and Gran Canaria (70.82%) (Table 2). This large natural parasitism rate suggests that *Trichogramma* parasitoids probably have a high affinity for *Ch. chalcites* eggs and their association with the banana as host plant in the Canary Islands. Five species of the genus *Trichogramma* were identified: *T. achaeae* Nagaraja and Nagarkatti, *T. bourarachae* Pintureau and Babault, *T. canariensis* del Pino and Polaszek, *T. euproctidis* Guirault and *T. evanescens* Westwood [31,32,33]. Among them, *T. achaeae* was found on all surveyed islands on banana and other host plants (Table 5), indicating its high potential as a biological control agent of *Ch. chalcites* in the Canary Islands [34]. According to the literature, other *Trichogramma* species have been cited parasitizing *Ch. chalcites* eggs with similar parasitism levels. For example, *T. cordubensis* Vargas and Cabello has been recorded as an important biological control agent in several biotopes of the Madeira Island [30] and tomato fields in Azores [29]. In banana plantations of the Ivory Coast, *T. lutea* Girault was found achieving a high parasitism rate of 80% [21]. Cabello [22] also found several *Trichogramma* species parasitizing eggs of noctuids on alfalfa, corn, cotton, and soybean fields in the south of Spain. In Catalonia, egg parasitism rates by *Trichogramma* spp. reached 80% in tomato crops [47].

However, surveys conducted to determine the action of larval/pupal parasitoids on *Ch. chalcites* populations showed a lower number of species than those described by Vilardebo and Guerout [21], but similar to those obtained by several authors in mainland Spain [22,45,46]. In order of importance the following parasitoids were identified: *Cotesia* sp. (Hym.: Braconidae), *Aplomyia confinis* Fallén (Dip.: Tachinidae), *Hyposoter rufiventris* Perez, *Ctenochares bicolorus* L. (Hym.: Ichneumonidae), *Cotesia glomerata* L. (Hym.: Braconidae) and *Aleiodes* sp. (Hym.: Braconidae). Some of these species have been previously cited as larval parasitoids of *Ch. chalcites* in the Canary Islands [37]. However, this is the first record of *A. confinis* and *H. rufiventris* as larval parasitoids of *Ch. chalcites* in the Canary Islands (Table 4). 

The most important larval parasitoid was an unidentified solitary species of the genus *Cotesia* Cameron (Hymenoptera: Braconidae) recorded in numerous localities of Tenerife and La Palma (Table 5). Several *Cotesia* species are of interest as biological control agents of pest caterpillars [48]. However, despite the economic and scientific importance of *Cotesia*, little is known about its systematics [49]. In the Spanish mainland, two species, *C. plutella* (Kurdjumov) and *C. kazak* (Telenga) have been cited as the most predominant native parasitoids of *Ch. chalcites* in outdoor and greenhouse crops [22,45,46]. Three species belonging to this genus have been previously recorded in the Canary Islands: *C. cuprea* (Lyle), *C. glomerata* L. and *C. vanessae* (Reinhard) [50]. Among them, the microgastrine wasp *C. glomerata* is a gregarious endoparasitoid of several species of *Pieris* (Lep.: Pieridae), such as *P. brassicae* L. and *P. rapae* L., which are important pests of cabbage crops (*Brassica oleracea* L.) [51]. However, this larval parasitoid has been described as an invasive species in La Palma attacking the large, white butterfly *Pieris cheiranthi* Hübner 1808, an endemic species closely associated with the local endemic ecosystems of relicts of the laurel forests [52]. 

In our surveys, an unidentified species of the genus *Aleiodes* Wesmael (Hymenoptera: Braconidae) was occasionally found parasitizing young larvae of *Ch. chalcites*. *Aleiodes* species are koinobiont, synovigenic and nearly strictly solitary endoparasitoids of Macrolepidopteran larvae (especially Noctuidae and Geometridae) [53]. For this reason, the members of this group are potentially very important biological control agents for several lepidopteran pests. According to Guerrero and Koponen [54], four species of the genus *Aleiodes* are present in the Canary Islands: *A. basalis* (Costa), *A. borealis* (Thomson), *A. ductor* (Thunberg) and *A. gastritor* (Thunberg), although their hosts’ identity is unknown. However, two unidentified species of this genus have previously been reported as biological control agents of *Ch. chalcites* and other Plusiinae species in horticultural protected crops in the Oeste region of Portugal [55].

The tachinid *A. confinis* is the only species of the genus *Aplomyia* present in the Canary Islands [50]. It is an endoparasitoid widely distributed throughout central and southern Europe, attacking lycaenid butterfly caterpillars [56]. In addition, another two tachinid species, *Exorista sorbillans* Wiedemann and *Drino inerbis* Wiedemann, have also been cited as larval parasitoids of *Ch. chalcites* in the Canary Islands [37] and Ivory Coast [21]. 

Among the ichneumonids, *H. rufiventris* is an endemic koinobiont endoparasitoid present throughout the archipelago [50]. A closely related species, *H. didymator* (Thunberg), has been identified as the most common native biocontrol agent of some noctuid species in Spain [22,57] and several European countries [58]. Finally, *Ctenochares bicolorus* is a large ichneumonid, widely distributed from Africa and southern Europe, which has been described above as a pupal parasitoid of *Ch. chalcites* [59].

In addition to the mortality caused by parasitoids, 32.07% of the collected *Ch. chalcites* larvae died by other causes (Table 3). Such causes include among others a native baculovirus of the genus Nucleopolyhedrovirus, previously described in banana crops in the Canary Islands as ChchNPV-SP2 [38]. 

According to our results, larval parasitism of *Ch. chalcites* was higher in open field (14.63%) than in greenhouses (5.08%). These low levels of larval parasitism in banana greenhouse plantations may be conditioned by: (1) climatic conditions, (2) greenhouse structures that prevent the access of parasitoids to the crop, (3) agronomic practices such as the repeated application of broad-spectrum pesticides, which are harmful to beneficial arthropods [12], and (4) a low initial incidence of *Ch. chalcites* populations [5]; similar situations have been described for greenhouse horticultural crops in southern Spain [46]. In addition, our open field larval parasitism rates were lower than the 36.3% recorded by Vilardebo and Guérout [21] in banana crops of Guinea and Ivory Coast.

Results of the present work indicate that populations of native parasitoids can exert a certain natural control of *Ch. chalcites*. However, their naturally occurring populations are insufficient to minimize the damage caused by this pest to below economic injury levels. Thus, it is necessary to develop IPM programs based on the use of selective insecticides, conservation of natural enemies and augmentative releases of mass-reared wasps [13,14,15]. According to our results on the percentage of parasitism, seasonal occurrence and host specificity, the parasitoid species *T. achaeae* and *Cotesia* sp. would be good candidates for biological control agents of *Ch. chalcites* in banana crops. However, this must be confirmed with other studies on several biological aspects of these parasitoid species. Currently, *T. achaeae* is commercialized and used in some European and North African countries against *Tuta absoluta* (Meyrick) (Lepidoptera: Gelechiidae) [41] and other Lepidoptera species in more than 15 horticultural and ornamental crops [60]. In the case of larval parasitoids of the genus *Cotesia*, some studies confirm the possibilities for the use of *C. marginiventris* (Cresson) as a biological control agent of *Ch. chalcites*, achieving parasitism levels of 100% [28,61]. However, refinement and improvement of mass rearing methods of this species are needed [62]. 

In the greenhouse trial, *T. achaeae* was shown to be a subtropical species well adapted to warm climates [63] and to the extreme temperatures (maximum and minimum) prevailing in banana greenhouses in the Canary Islands, as previously observed by Cabello et al. [41] in southern Spain. However, extreme temperatures have been reported to have a harmful impact on the biology and parasitization rates of *T. achaeae* [34] and other *Trichogramma* species [64,65].

The highest parasitism levels observed in the greenhouse bioassay exceeded 82% when *T. achaeae* was released at a rate of 35 adults/m^2^ every 21 days, which should provide excellent pest control (Figure 4). The incidence of parasitism found in the current study was like those reported for other lepidopteran pests using different *Trichogramma* species in several crops [66,67] (p. 424). Pizzol et al. [26] reported a similar result using *T. evanescens* for the control of *Ch. chalcites* in tomato greenhouse crops in semi-field conditions, performing three releases of nine individuals/m^2^ every 15 days. These application rates appear to be high but are within what is commonly used in inundative biological control programs in other crops [68]. 

Populations of *Ch. chalcites* larvae on banana plants in the *T. achaeae* release and chemical control plots remained at similar levels, with an average of 1.54 and 1.41 larvae/plant, respectively (Figure 5). However, the mean foliar damage level was lower in the *T. achaeae* release plot (17.07%) relative to the chemical control plot (20.26%) (Figure 6), which indicates that the strategy is effective. It is important to underline the high mean parasitism rates recorded in the chemical control plot (47.25%), due to the natural dispersion of *T. achaeae* from the biological control plot, which contributed positively to the control of the pest. The above data indicate that the trial design was not the most appropriate. However, it should be noted that since the trial was carried out in a commercial greenhouse, the two main plots could not be isolated. This high natural dispersal ability of *Trichogramma* parasitoids from the release points has been studied by several authors [69,70] and depends on the number of release points, plant structure and wind, among other factors [71].

The reported data show the potential of *T. achaeae* to control *Ch. chalcites* in banana plantations. In our case, *T. achaeae* was released on egg cards at the rate of 35 adults/m^2^ (140 cards per ha). Considering that an average of four releases (spaced 21 days apart) were required and that the average cost of a card is 1 €, the total cost of *Ch. chalcites* control during a cycle of banana cultivation in the Canary Islands would likely average 560 €/ha. This cost is much higher than the average cost of indoxacarb (240 €/ha), which is the most frequently used pesticide in banana crops [6]. Therefore, it is necessary to carry out other studies to establish the most appropriate release doses under controlled conditions to obtain consistent results and reduce the costs for release of *T. achaeae* to control *Ch. chalcites*. In addition, the effectiveness of *T. achaeae* may also be influenced by the optimum introduction times of the parasitoid according to (1) the density or phenology of the pest, (2) quality of the parasitoids released, (3) method of distribution, (4) number of release points, (5) crop phenology, (6) intra-guild interactions with other biological control agents (polyphagous predators), and (7) use of insecticides into the parasitoid release areas [67,68] (pp. 191–208). However, these results support a strategy for the biological control of *Ch. chalcites* based on the periodical inundative releases of *T. achaeae* when the first flights of adults and eggs of the pest are detected in the crop [5]. These inundative releases will be a complement to the high natural parasitism that arrives later to the fields [33]. Likewise, to obtain a high control of the pest, one strategy may be to combine releases of *T. achaeae* with the application of biorational products [6,11], such as *Bacillus thuringiensis* subsp. *kurstaki*, alphabaculovirus (ChchNPV-TF1) or Indoxacarb, for the control of young *Ch. chalcites* larvae. This strategy has been successfully employed for the control of *T. absoluta* in Spain [41] and is considered a viable tactic in integrated pest management. Other aspects such as the use of ecological infrastructures (cover plants, flower strips, weedy margins, and hedges), which maintain and enhance the reproduction, survival and efficacy of natural enemies present in the banana agroecosystems are also recommended to control *Ch. chalcites* [72].

## Figures and Tables

**Table 1:** Parasitism of *Chrysodeixis chalcites* eggs collected on banana crops in the Canary Islands.

Island		No. of Eggs		Parasitism (%)
	Collected	Non-Parasitized	Parasitized	
El Hierro	53	18	35	66.04
Gran Canaria	248	112	136	54.84
La Palma	1097	109	988	90.06
Tenerife	1370	702	668	48.76
Total	2776	944	1785	64.30

**Table 2:** Relative importance of parasitized eggs of *Chrysodeixis chalcites* per field in banana crops of the Canary Islands.

Island	No. Fields	No. Eggs per Field		Parasitism per Field (%)	
		Sampled	Parasitized	Average	Maximum
El Hierro	7	7.57 ± 11.90 c	5.00 ± 10.57 c	48.57 ± 11.27 ab	100.00
Gran Canaria	2	58.50 ± 22.27 a	41.50 ± 19.78 a	70.82 ± 21.08 ab	71.64
La Palma	22	49.14 ± 6.71 b	42.05 ± 5.96 a	81.01 ± 6.36 a	100.00
Tenerife	28	48.93 ± 5.95 b	23.86 ± 5.28 b	38.58 ± 5.63 b	97.06
Total	59	44.42 ± 4.38	29.00 ± 3.89	56.68 ± 4.59	100.00

Means in each column followed by the same letter are not significantly different, *p* < 0.05, Tukey’s HSD test.

**Table 3:** Parasitism of noctuid pest larvae collected on banana crops in the Canary Islands.

Banana Field System	Noctuid Species	No. of Larvae			Parasitism (d)
		Collected (a)	Died (b)	Parasitized (c)	(d = 100c/a − b)
Open field	*Ch. Chalcites*	1203	376	121	14.63
	*C. circunflexa*	92	10	10	12.20
	*S. exigua*	5	0	0	0.0
	*S. littoralis*	62	15	0	0.0
	*T. orichalcea*	13	0	0	0.0
Greenhouse	*Ch. Chalcites*	4276	1381	147	5.08
	*C. circunflexa*	3	0	0	0.0
	*S. exigua*	42	6	0	0.0
	*S. littoralis*	226	48	7	3.93
Total		5922	1836	285	6.98

**Table 4:** Relative importance, range and host stage of larval noctuid parasitoid on banana crops in the Canary Islands.

Parasitoid	Host Plant	Host		No. Fields ^2^	Parasitism per Field (%)	
		Species	Stage at Sampling ^1^		Average	Maximum
*Aplomyia confinis*	Geranium	*Ch. chalcites*	L2–L5	1	5.26	5.26
	Banana	*Ch. chalcites*	L2–L5	16	4.22	10.35
*Cotesia glomerata*	Banana	*Ch. chalcites*	L3–L4	2	3.01	3.70
*Cotesia* sp.	Squash	*Ch. chalcites*	L2–L4	1	16.67	16.67
	Geranium	*Ch. chalcites*	L2–L4	1	12.50	12.50
	Bean	*Ch. chalcites*	L2–L4	2	5.33	7.14
	Potato	*Ch. chalcites*	L2–L4	1	4.17	4.17
	Pepper	*Ch. chalcites*	L2–L4	1	12.50	12.50
	Banana	*Ch. chalcites*	L2–L4	11	8.18	19.23
	Tomato	*Ch. chalcites*	L2–L4	6	13.02	33.33
	*N. glauca*	*C. circunflexa*	L2–L4	1	4.55	4.55
*Ctenochares bicolorus*	Banana	*Ch. chalcites*	L3–P	3	4.60	6.89
*Hyposoter rufiventris*	Geranium	*Ch. chalcites*	L2–L4	1	5.26	5.26
	Potato	*Ch. chalcites*	L2–L4	1	1.96	1.96
	Banana	*Ch. chalcites*	L2–L4	5	2.18	3.49
	Banana	*S. littoralis*	L2–L4	2	17.19	25.00
	Tomato	*Ch. chalcites*	L2–L4	3	7.42	8.33
*Aleiodes* sp.	*N. glauca*	*Ch. chalcites*	L2–L3	1	4.76	4.76
	Tomato	*Ch. chalcites*	L2–L3	1	4.88	4.88

^1^ L: larva (L1–L6 instars); P: pupa. ^2^ No. of sampled fields in which specimens of noctuid species were collected.

**Table 5:** Parasitoid species of *Chrysodeixis chalcites* found in the surveyed banana production areas located in the Canary Islands from 2007 to 2010.

Species	Year and Island ^a^				Field System ^b^	
	2007	2008	2009	2010	Openfield	Greenhouse
Larval/Pupa parasitoids						
Braconidae						
*Cotesia* sp.		TF ^3^, LP ^9,10,12^	TF ^1,2,3,5,6,7^, LP ^10^		Ba, To, Sq, Be, Ge, Ng	Ba, Ca, Pe, Po
*Cotesia glomerata* (Linnaeus, 1758)		TF ^3^, HR ^14^			Ng	Ba
*Aleiodes* sp.		HR ^14^	TF ^5^		To, Ng	
Ichneumonidae						
*Ctenochares bicolorus* (Linnaeus, 1767)	TF ^3^	TF ^3^	TF ^1^, LP ^9^		Ba	Ba
*Hyposoter rufiventris* (Pérez, 1895)	TF ^7^	TF ^3^, HR ^14^	TF ^3,5^, LP ^12^		Ba, To, Ge	Ba, Po
Tachinidae						
*Aplomyia confinis* (Fallén, 1820)	TF ^2,5^	TF ^3^	TF ^1^, LP ^8,12^		Ba, Ge	Ba
Egg parasitoids						
Trichogrammatidae						
*Trichogramma achaeae* Nagaraja and Nagarkatti, 1970		TF ^3^, LP ^9,10^, HR ^14^	TF ^1,4,5,7^, LP ^8,9,10,11^, HR ^14^, GC ^13^	LP ^9^	Ba, To, Ca, Be, Ng, Sn	Ba, Pe, Ca, Sq
*Trichogramma bourarachae* Pintureau and Babault, 1988			TF ^4,7^		Ng	
*Trichogramma canariensis* del Pino and Polaszek, 2013			GC ^13^	GC ^13^	Ba, To	Ba
*Trichogramma euproctidis* (Guirault, 1911)		TF ^3^	TF ^1,3,4,5,7^		Ba, To, Ng, Sn	Ba, To, Ng
*Trichogramma evanescens* Westwood, 1833			TF ^1,4,6^, LP ^9^		To, Ng	Ca, Sq

^a^ Island: El Hierro (HR), Gran Canaria (GC), La Palma (LP), Tenerife (TF). ^b^ Plant species: banana (Ba), squash (Sq), cabbage (Ca), geranium (Ge), bean (Be), pepper (Pe), tomato (To), potato (Po), *Nicotiana glauca* (Ng) and *Solanum nigrum* (Sn). Superscripts on the island acronyms indicate the locality where the parasitoid species was found: 1, Caldera del Rey; 2, Cueva del Polvo; 3, Las Galletas; 4, Guía de Isora; 5, Hoya Meleque; 6, Pajalillos; 7, Valle de Guerra; 8, Breña Baja; 9, El Remo; 10, Fuencaliente; 11, Los Llanos de Aridane; 12, Puerto de Tazacorte; 13, Arucas; 14, Frontera.

## Data Availability

The data presented in this study are available on request from the corresponding author.

## Supplementary Materials

The following supporting information can be downloaded at: https://www.mdpi.com/article/10.3390/insects13060516/s1, Table S1: Collection localities of *Chrysodeixis chalcites* parasitoid species in the Canary Islands.
