# Supplementary material for: Biological Control Options for the Golden Twin-Spot Moth, Chrysodeixis chalcites (Esper) (Lepidoptera: Noctuidae) in Banana Crops of the Canary Islands
Source: Insects. 2022 May 31;13(6):516. doi: 10.3390/insects13060516 (PMC9225108; doi:10.3390/insects13060516)
Supplement: Supplementary file 1 [file insects-13-00516-s001.zip › insects-1734595-supplementary.pdf]

Table S1. Collection localities of *Chrysodeixis chalcites* parasitoid species in the Canary Islands.

Island	Locality	Coordinates	
		**Long. (W)**	**Lat. (N)**
Tenerife	Caldera del Rey	16º 43′ 15″	28º 04′ 31″
	Cueva del Polvo	16º 49′ 57″	28º 13′ 44″
	Las Galletas	16º 39′ 32″	28º 01′ 52″
	Guía de Isora	16º 48′ 34″	28º 11′ 22″
	Hoya Meleque	16º 33′ 31″	28º 24′ 22″
	Pajalillos	16º 23′ 13″	28º 31′ 42″
	Valle de Guerra	16º 23′ 07″	28º 32′ 26″
La Palma	Breña Baja	17º 45′ 31″	28º 36′ 44″
	El Remo	17º 53′ 17″	28º 33′ 21″
	Fuencaliente	17º 51′ 42″	28º 28′ 43″
	Los Cancajos	17º 45′ 37″	28º 39′ 28″
	Los Llanos de Aridane	17º 55′ 53″	28º 37′ 27″
	Puerto de Tazacorte	17º 56′ 44″	28º 39′ 48″
Gran Canaria	Arguineguín	15º 40′ 28″	27º 45′ 45″
	Arucas	15º 30′ 54″	28º 07′ 58″
	Gáldar	15º 39′ 49″	28º 09′ 26″
	Vecindario	15º 25′ 44″	28º 07′ 52″
El Hierro	Frontera	38º 00′ 28″	27º 46′ 51″
